# Altered CD4 T cell response in oligometatastic non-small cell lung cancer brain metastasis

**DOI:** 10.1186/s40478-025-02011-1

**Published:** 2025-05-09

**Authors:** Mais Alsousli, Cecile L. Maire, Andras Piffko, Jakob Matschke, Laura Glau, Merle Reetz, Svenja Schneegans, Gresa Emurlai, Benedikt Asey, Alessandra Rünger, Sven Peine, Jolanthe Kropidlowski, Jens Gempt, Markus Glatzel, Manfred Westphal, Eva Tolosa, Katrin Lamszus, Klaus Pantel, Simon A. Joosse, Malte Mohme, Harriet Wikman

**Affiliations:** 1 Department of Tumor Biology, University Medical Center, Hamburg-Eppendorf, Hamburg, Germany; 2 Department of Neurosurgery, University Medical Center Hamburg-Eppendorf, Hamburg, Germany; 3 Institute of Neuropathology, University Medical Center Hamburg-Eppendorf, Hamburg, Germany; 4 Institute of Immunology, University Medical Center Hamburg-Eppendorf, 20246 Hamburg, Germany; 5 Institute of Transfusion Medicine, University Medical Center Hamburg-Eppendorf, Hamburg, Germany; 6 Mildred Scheel Cancer Career Center HaTriCS4, Hamburg, Germany; 7 Institute of Tumor Biology, University Medical Center Hamburg-Eppendorf, Martinistrasse 52, 20246 Hamburg, Germany

**Keywords:** NSCLC, Brain metastasis, Immunophenotyping, T cells, CD4

## Abstract

Lung cancer is the leading cause of cancer mortality globally, with brain metastasis (BM) in 40% of advanced non-small-cell lung cancer (NSCLC) cases. In 15% of these cases, the brain is the sole affected organ (oligometastasis), which correlates with a better prognosis compared to widespread disease. It remains unclear if brain-only metastasis without systemic spread is due to the immune system’s ability to control systemic tumor progression. We studied the immune cell compositions in NSCLC patients with BM, identifying novel patterns associated with BM, and specifcally with either oligo- or polymetastatic spread. Multi-parametric immune phenotyping of peripheral blood primarily showed alterations in the CD4^+^ T cell compartment, with increased CD4^+^ T_H_17 cells, and higher IL-17 levels in NSCLC BM patients compared to healthy individuals. Furthermore, CD4^+^ T cells in BM patients exhibited lower CD73 expression and reduced effector memory differentiation. There was also decreased intratumoral infiltration and a distinct CD4^+^ T cell profile in oligo-synchronous BM, both in the tumor microenvironment and peripheral blood, compared to polymetastatic BM patients. Additionally, CD73 was significantly upregulated in CD4^+^ and T regulatory cells of oligo-synchronous (BM simulantously with primary-tumor diagnosis) BM. These findings suggest that CD4^+^ T cells play a crucial role in the biology of NSCLC BM and potentially contribute to differences in metastatic patterns, as oligo-synchronous BM shows a more significant alteration in the CD4^+^ T cell immune profile, both locally at the tumor site and systemically.

**Supplementary Information:**

The online version contains supplementary material available at 10.1186/s40478-025-02011-1.

## Introduction

Lung cancer is the leading cause of cancer-related mortality worldwide, accounting for more deaths than colon, breast, and prostate cancers combined [1]. This high mortality rate is attributed to early and rapid metastasis to multiple organs, with the brain being affected in approximately 40% of patients with advanced non-small cell lung cancer (NSCLC) patients [2].Consequently, brain metastases (BM) have become a significant limitation in the life expectancy and quality of life of many individuals. Therefore, the development of effective management strategies for BM is of paramount importance.

A distinctive feature of NSCLC is the frequent occurrence of oligometastatic brain disease. Indeed, more than 15% of all metastatic lung adenocarcinomas exclusively affect the brain as the sole organ involved, either at the time of primary diagnosis (oligo-synchronous) or after primary diagnosis (oligo-metachronous) [3]. Clinical data suggest that oligo-brain metastatic disease, often associated with a favorable prognosis, represents a distinct form of cancer spread compared with a disease that disseminates uncontrollably to multiple sites (polymetastatic) [4–6]. Several prospective studies have demonstrated significantly improved progression-free (PFS) or overall survival (OS) in oligometastatic NSCLC patients who received stereotactic body radiotherapy compared with maintenance therapy alone [7–9].

The metastatic spread of primary tumors is neither random nor influenced solely by anatomical location, whereby host-related elements, such as immunological properties and target organ microenvironment, are hypothesized to play a crucial role regulating these processes [3]. A dysregulated immune system is widely recognized as a hallmark of cancer, in which both positive and negative interactions between immune and neoplastic cells play a significant role in malignant progression [10]. Due to the unique properties and immune context of the brain microenvironment [11, 12], the phenotype and activation pattern of immune cells may differ in the tumor microenvironment as well as in the peripheral blood when they metastasize to the brain. In the present study, we aimed to discern T cell populations in both brain metastatic tissue and peripheral blood to elucidate the immunological landscape in NSCLC patients with BM, and to identify novel immune patterns related to the occurrence of brain metastases, either oligo- or polymetastatic disease. Beyond understanding the biological basis for different metastatic patterns in BM, such approaches can provide new insights into immunomodulatory treatment strategies targeting specific metastasis-induced inflammatory states.

## Materials and methods

### Patient cohorts

All patients included in this study underwent surgery for lung cancer BM at the Department of Neurosurgery at the University Hospital Hamburg-Eppendorf (UKE) between 2014 and 2020. Patients with known driver mutations in *EGFR*, *ALK*, or *ROS* were excluded from this study. The clinical characteristics of the 69 patients with NSCLC are summarized in Table 1. Of these, 54 patients were included in the peripheral blood analyses and 56 in the tissue phenotyping, including 28 non-overlapping samples (Supplemental Tables 1 and 2). This study was approved by the local ethics council of the Hamburg chamber for physicians under numbers Nr. PV5392 & Nr. PV4904 and was performed in accordance with the Helsinki Declaration of 1975.

**Table 1:** Patient characteristics

Characteristics		All BM	Oligo-sync.	Oligo-metac.	Poly-met.	*P*-value
Samples n (%)		69	23 (33.3)	20 (29)	26 (37.7)	0.368
Gender n (%)	F	38 (55.1)	10 (43.5)	11 (55)	17 (65.4)	0.331
	M	31 (44.9)	13 (56.5)	9 (45)	9 (34.6)	
Age at PD (y)	range	33–78	45–78	34–76	33–77	0.48
	mean	59.3	61.2	59.2	57.7	
Age at BM-OP (y)	range	34–83	45–83	34–78	35–78	0.422
	nean	60.2	61.6	61.3	58.1	
Histology, n (%)	AC	63	21	16	26	0.141
	SQ	3	1	2	0	
	LCNEC	3	1	2	0	
Time btw PD & BM OP (months)	range	0–69	0	2–69	0–30	0.965
	mean	10.2	0	24.3	5.4	
Neoadjuvant treatment	none	40	22	1	17	**< 0.0001**
	IT	4	0	1	3	
	RCT	25	1	18	6	
Survival	alive	20	6	4	10	0.380
(m)	dead	49	17	16	16	
FUP after BM OP (m)	range	0–83	2–65	0–59	0–83	0.678
	mean	17.7	20.2	17.4	15.2	

BM: brain metastases, PD: Primary diagnosis, BM-OP: brain metastases operation, F: female. M: male, AC: adenocarcinoma, SQ: Squamous cell carcinoma, LCNEC: Large cell neuroendocrine carcinoma, TN: treatment-naive, IT: immunotherapy, RCT: Radio-/Chemotherapy

### Immunohistochemical assays

Immunohistochemical staining (IHC) was performed on 2 μm thick sections of formalin-fixed, paraffin-embedded (FFPE) tissue samples of BM (*n* = 56) from the Department of Neuropathology, UKE). Antibodies used for IHC were anti-CD3 (clone SP7; 1:100; M3074, Zytomed), anti-CD8 (clone SP16; 1:500; C1008C01, DCS), anti-CD4 (clone 4B12; 1:50; M731001-2, DAKO), anti-FOXP3 (clone D2W8E; 1:100; 98377, Cell Signaling), anti-CD68 (clone KP1; 1:100; M087629-2, DAKO), and Ki67 (clone SP6; 1:750; 275R-15, Cell Marques). All staining was performed on a Ventana benchmark XT autostainer following the manufacturer’s recommendations. Percentages of positively stained cells were evaluated in two regions: intratumoral (within the solid tumor tissue area) and peritumoral (the adjacent area around the tumor tissue). Immunohistochemical evaluation was performed semi-quantitatively by a pathologist as described earlier [13], briefly, for CD3, CD8, CD4, CD68, and Ki67, a score of 0 (negative: no stained cells), 1 (low: <10%), 2 (moderate:10–40%), or 3 (high > 40%) was given based on the estimation of positively stained immune cells out of total cells in the investigated intratumoral or peri-tumoral region (*n* = 56). For statistical analysis, moderate and high patients were combined (as high), negative, and low (as low). Because the number of FoxP3^+^ cells was relatively low in all BM groups, samples were scored as negative (0%) or positive (≥ 1%) in both intratumoral and peritumoral regions (*n* = 60).

### Immune cell isolation

Peripheral blood was collected into 7.5 ml EDTA-containing tubes before surgical removal of the tumor. In addition to the 54 patients, control samples were obtained from 20 anonymized, age-matched healthy donors (HD) from the Department of Transfusion Medicine, University Hospital Hamburg-Eppendorf (Hamburg, Germany). PBMC were isolated within 2 h of blood collection using Ficoll^®^ gradient centrifugation as described before [14] and stored in RPMI/10% DMSO (P04-17500) at -80 °C until further use.

### Multicolor flow cytometry

Frozen PBMCs were thawed in a water bath at 37 °C, washed with 4 °C cold 10% FBS in RPMI, resuspended in a cold medium (RPMI, 10% FCS and DNAse (1:1000) (4 °C), and counted using Vi-CELL^®^ XR Cell Viability Analyzer (383556, Beckman Coulter). Five different panels (43 antibodies) were used to analyze T cell exhaustion, T cell differentiation, T helper cell subsets, T cell metabolism, and cytokine secretion (Supplemental Table 3).

For cell surface staining, samples were resuspended in flow cytometry staining buffer (eBioscience) with Fc-block (Miltenyi Biotec) and stained with the antibody cocktails at room temperature for 45 min in the dark. For intracellular staining (cytokines), after 4 h of incubation at 37 °C and 5% CO_2_, a stimulation mix (Phorbol 12-Myristate 13-Acetate (PMA) (1 µg/ml; P1585, Sigma), ionomycin calcium salt (1 mg/ml; 10634, Sigma), Brefeldin A Solution 1000 × (3 mg/ml; 00-4506, Invitrogen), and X-Vivo 15 serum-free (881024, Biozym) was added to each sample, and cells were incubated again for another 5 h at 37 °C and 5% CO_2_. The samples were then washed, resuspended in flow cytometry staining buffer, and stained with a surface antibody cocktail for 10 min in the dark. After further washing, the cells were fixed and permeabilized before staining with the cytokine (intracellular) antibody cocktail for 30 min in the dark at room temperature. Following incubation, the cells were washed and resuspended in flow cytometry staining buffer. The analysis was performed using a BD LSR Fortessa flow cytometer. Data were exported as.fcs files and manual gating was carried out using the FACSDiva software (version 9.1 Becton Dickinson). The gating strategies are presented in Supplementary Figs. 1–4. Samples with data from less than 1000 living T cells were excluded from the analyses.

### Statistical analysis

Data analysis was performed using In-Silico Online v2.3.1, R version 4.1.3 (http://in-silico.online), and GraphPad Prism Software (v9.5.0, GraphPad Inc., Boston, MA, USA). Associations between independent nominal data were calculated using Fisher’s exact test, whereas those between dependent data were assessed using McNemar’s test. Kruskal-Wallis and Wilcoxon’s tests were performed to calculate the significance of the differences between medians, and the significance of the mean difference was calculated using ANOVA. An alpha level of 0.05 was used to determine statistical significance and was corrected for multiple testing where appropriate.

For flow cytometry panel 2, we performed a UMAP dimensionality reduction and unsupervised clustering analysis. Compensated and normalized data containing clean CD3^+^ cells were used to generate UMAP dimensionality reduction (using the R-package “umap”) and flowClust clustering of all groups merged (using the R package “flowClust”). The marker expression heatmap was calculated as the median fluorescence intensity of each marker per flowClust cluster. Comparisons between groups were performed using a t-test. Language editing and spell checks were performed using Paperpal software.

## Results

### Patients’ characteristics

Sixty-nine NSCLC patients with BM were recruited for this study (Table 1 and Supplemental Tables 1 and 2). The patients were divided into two main groups: (1) NSCLC patients with BM only (oligometastatic; lymph node dissemination including mediastinal lymph node involvment allowed) and (2) NSCLC patients with metastases in the brain and other extra-cranial organs (poly metastatic). The oligometastatic group was further subdivided into: 1a) patients with brain metastasis at the time of primary diagnosis (synchronous oligometastasis) and 1b) patients with brain metastases after primary diagnosis (metachronous oligometastasis). Regarding the main clinical determinants of the three groups, neoadjuvant treatment was the only significant difference between the groups (*P* < 0.0001, Table 1).

### Immune profiling of NSCLC brain metastasis

**Fig. 1:**
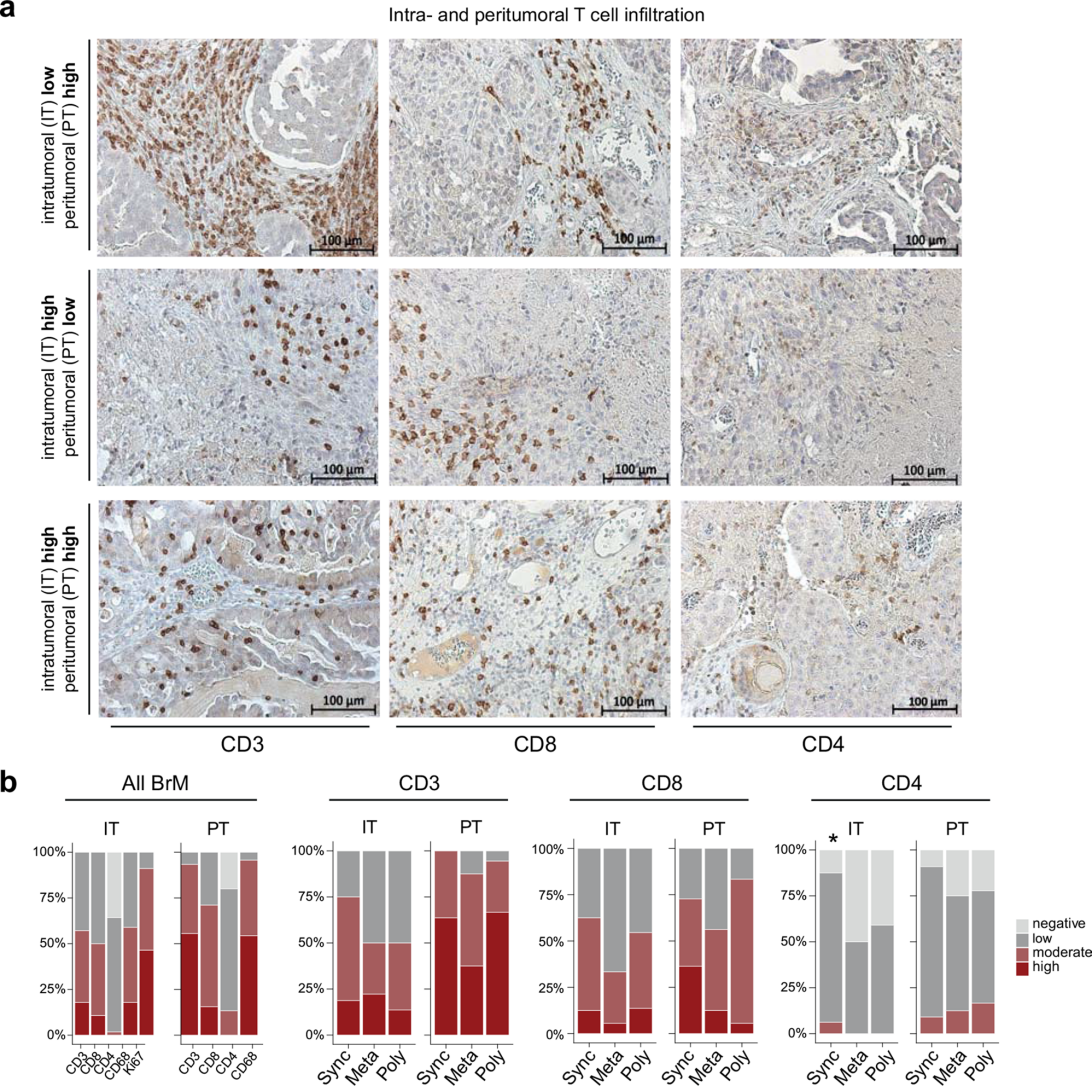
(**a**.) Immunohistochemical detection and estimation of positive staining for CD3, CD8, and CD4 on TILs in brain metastatic tissues. Representative stainings (high and low infiltration)are shown for both intra-tumoral (IT) and peri-tumoral (PT) regions. Magnification: x20. **(b.)** Comparison of the staining scores for CD3, CD8, and CD4 in all groups combined, and between oligo-synchronous BM (Sync), oligo-metachronous BM (Meta), and poly BM (Poly) individually in both IT and PT regions. BM: brain metastasis, Sync: oligo-synchronous BM, Meta: oligo-metachronous BM, and poly: poly metastasis, negative: no stained cells, low: <10%, moderate: 10–40%, and high: >40%

To establish a brain metastasis-associated immune profile, the spatial distribution of infiltrating immune cells was evaluated in the peritumoral and intratumoral regions of BM tissues from patients with NSCLC by immunohistochemical (IHC) staining for CD3, CD8, CD4, FoxP3, and CD68 (Fig. 1a and Supplementary Fig. 5). In addition, tumor cell proliferation was determined by Ki67 staining (Supplementary Fig. 5).

Overall, immune infiltration was more commonly found in the peri-tumoral area than in the core of the BM (intra-tumoral), with a significantly higher frequency of CD3^+^ T cell infiltration and CD68^+^ microglia/macrophages in the peri-tumoral regions (93.3 and 95.7% moderate/high inflation, respectively) compared to intra-tumoral regions (57.1 and 58.9%) (*P* < 0.0001 for both markers, Fisher’s exact test). Furthermore, within the CD3^+^ T cell population, a significantly greater proportion of CD8^+^ cells with moderate and high infiltration, was observed in both intratumoral and peritumoral lesions (50.0 and 71.1%, respectively) than in CD4^+^ cells (1.8 and 13.3%, respectively) (*P* < 0.0001 in both regions, McNemar’s test). The proliferation rate did not correlate with immune cell profiles (Fig. 1b). Taken together, different types of immune cells infiltrate the brain in NSCLC BM, however, these cells are found more commonly in the peri-tumoral area compared to intra-tumoral regions.

When analyzing oligo- and polymetastatic cases separately, IHC staining did not reveal any significant differences in the number of CD3^+^ or CD8^+^ cells between the groups in either region (Fig. 1b). However, patients with oligo-synchronous BM demonstrated a significantly higher infiltration of CD4^+^ T cells in the intratumoral region than poly- and oligo-metachronous BM patients (*P* = 0.044, G-test). Specifically, in the latter two groups, 50% and 60% of the samples had detectable CD4^+^ T-cells within the tumor tissues, whereas 87.5% of oligo-synchronous cases showed CD4^+^ T cell infiltration (Fig. 1b). No significant differences were observed in the number of microglia/macrophages (CD68^+^ cells) or Ki67-positive tumor cells among the three BM groups (Supplementary Fig. 5).

As the tumor-infiltrating CD4^+^ cell population was elevated in oligo-synchronous BM, we further investigated the frequency of regulatory T cells (T_regs_) defined by FoxP3 expression (Fig. 1a). Comparisons among the BM groups showed no statistically significant differences in T_regs_, either intratumoral or peritumoral (*P* = 0.524 and 0.831, respectively; Fisher’s exact test; Supplementary Fig. 6).

In summary, the immunophenotyping of the BM tissue, identified CD68^+^ cells to be the most prevalent immune cell type. We detected also in genereal, significantly more CD68^+^ cells and lymphocytes (TILs) in the peri-tumoral regions compared to the intra-tumoral regions. However, similar to other studies we found that the number of TILs was noticeably lower than what has been reported for primary lung tumors, indicating the presence of a more immunosuppressive microenvironment in the brain [15–17]. When the BM groups were analyzed sepratelly, a significantly increased CD4^+^ T cell BM inifiltration was detected among oligometastatic BM patients.

### Systemic Immunomodulation in patients with NSCLC compared to healthy donors

**Fig. 2:**
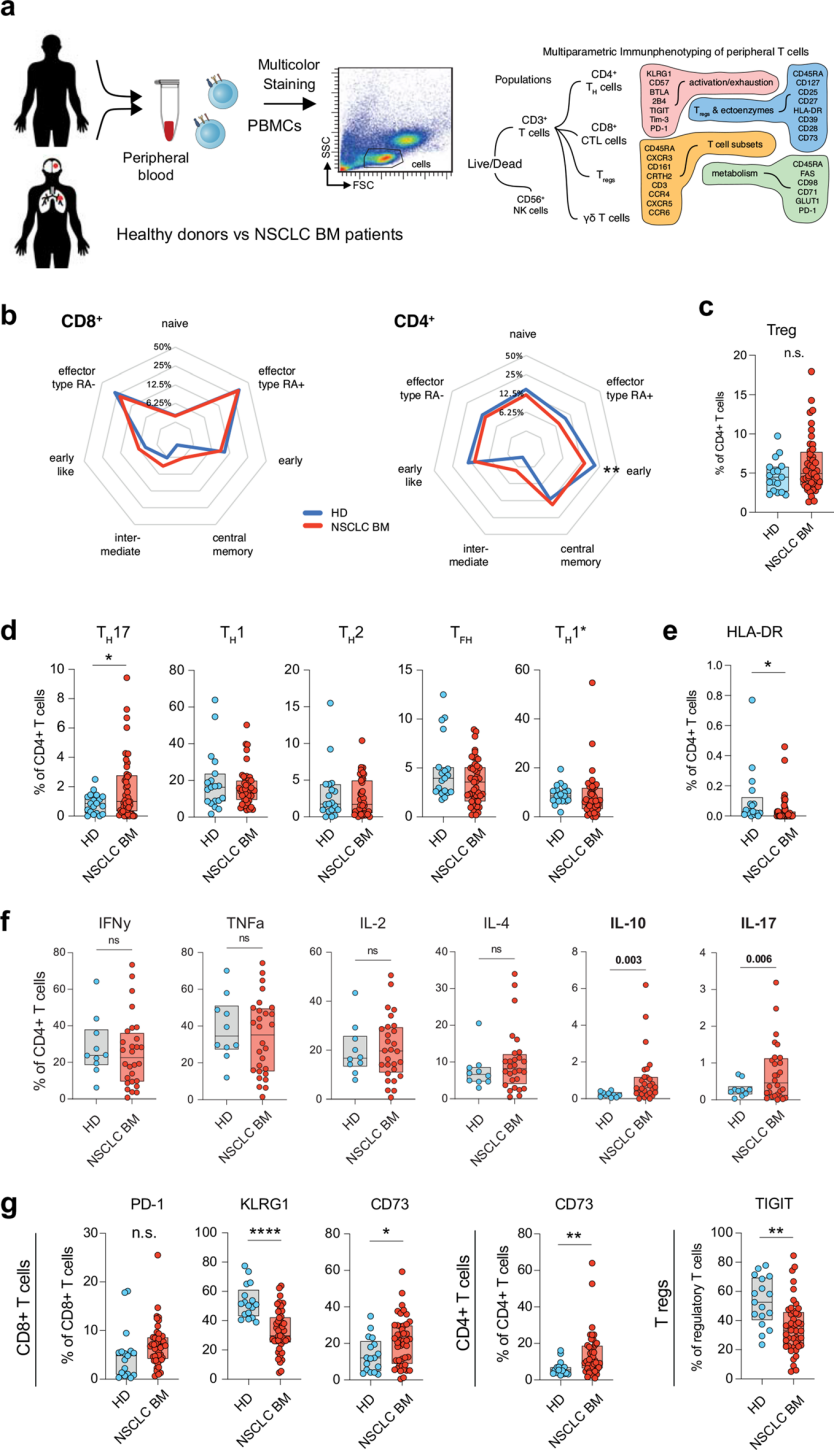
**(a)** Flow cytometric immunophenotyping of T cell activation and exhaustion marker expression of peripheral blood from NSCLC BM patients and age-matched healthy donors (HD). **(b)** Spider plots of CD4^+^ and CD8^+^ differentiation phenotype differences detected in peripheral blood of BM patients and HD. **(c)** Box plots depicting expression of T regs in HD and BM patients with no significant differences found. **(d)** A significantly higher frequency of pro-inflammatory T_H_17 T cells among cancer patients compared to HD was observed, **(e)** whereas a reduced HLA-DR expression did not support and activated phenotype in CD4^+^ T cells. **(f)** Box plots depicting expression of different cytokines IFN*γ*, TNF*α*, IL-2, IL-4, IL-10, and IL-17 on CD4^+^ and CD8^+^ cells. **(g)** Box plots for PD-1, KLRG1 and CD73 expression on CD8^+^ T cells, and TGIT expression on T regs. Mean ± SEM, t test. P values are defined as * < 0.05; ** < 0.01; and *** < 0.001

To investigate if differences in intratumoral CD4^+^ T cell infiltration were based on a differential immune activation pattern in the peripheral blood, we performed flow cytometry analyses on peripheral blood from patients with NSCLC BM and compared the T cell immunophenotypes to those of age-matched healthy individuals using five different multicolor antibody staining cocktails (Fig. 2a). First, we assessed the frequencies of the different stages of T cell development using CD45RA, CD27, CD28 and CCR7, defining seven differentiation states: Naïve: (CD45RA^+^, CD27^+^, CD28^+^, CCR7^+^), Central memory: (CD45RA^−^, CD27^+^, CD28^+^, CCR7^+^), Early memory (Early: CD45RA^−^, CD27^+^, CD28^+^, CCR7^−^), Early-like memory (Early-like: CD45RA^−^, CD27^−^, CD28^+^, CCR7^+^), Intermediate (CD45RA^−^, CD27^+^, CD28^−^, CCR7^−^), Effector memory RA^−^ (T_EM_) (effector memory cells: CD45RA^−^, CD27^−^, CD28^+/−^, CCR7^−^) and T Effector memory RA^+^ (T effectors type RA+: CD45RA^+^, CD27^−^, CD28^−^, CCR7^−^) [18]. While non significant differences were observed in the CD8^+^ T cell compartment, we found a significant decrease in early memory CD4^+^ T cells (Fig. 2b and Supplementary Fig. 7a), shifting towards more central memory and intermediate CD4^+^ subpopulations in the BM patients. No differences were found between circulating Tregs in healthy individuals and NSCLC BM patients (Fig. 2c).

Next, we analyzed the composition of peripheral CD4^+^ T helper cell subsets in NSCLC patients with BM compared to that in healthy individuals. While no differences between the study participants were observed in terms of the classical T_H_1 and T_H_2 subsets (Fig. 2d), we found a significantly higher frequency of pro-inflammatory T_H_17 T cells (CD4^+^, CD45RA^−^, CCR6^+^, CCR4^+^, CXCR3^−^, CD161^+^; *P* = 0.004, Welch’s t-test) among cancer patients compared to healthy individuals. However, although a proinflammatory T helper cell composition was observed, the reduced HLA-DR expression did not support and activated phenotype in CD4^+^ T cells (Fig. 2e).

To follow up on this finding, we also conducted intracellular cytokine staining of T cells. Among the NSCLC patients, we found that 14/28 (50%) of NSCLC BM patients showed reduced overall cytokine production (Supplemental Fig. 7b), while the other half of the patients could be divided into a group of predominantly TNFa and IFNy producers (9/14), and a second subgroup (5/14) of patients with polycytokine production. We found significantly higher frequencies of IL-10 in the CD4^+^ and CD8^+^ T cells of BM patients compared to healthy individuals (Fig. 2f; *P* = 0.003 and 0.015, respectively, Welch’s t-test). In agreement with our finding of increased T_H_17 CD4^+^ T helper cells, we also observed a significant increase in IL-17 production in CD4^+^ T cells (*P* = 0.006, Welch’s t-test). The finding of an increased IL-17 production is in line with our observation of increased activation of the T_H_17 axis, and thus a potentially pro-inflammatory peripheral immune environment in NSCLC patients with BM. No significant differences were observed in the T cell metabolism profile between patients with NSCLC and HD (data not shown).

To assess the expression of activation and exhaustion markers on the surface of peripheral blood T cells, we performed additional flow cytometry profiling. Here, the frequency of 5′-ectonucleotidase CD73, which converts adenosine monophosphate to immuno-inhibitory adenosine, expressed by CD8^+^ and CD4^+^ T cells, was significantly upregulated in cancer patients compared to that in healthy individuals (Fig. 2g, *P* = 0.022 and *P* = 0.0001, respectively, Welch’s t-test). No significant difference was observed in the frequency of cells expressing the classical immune checkpoint PD-1, although a strong trend in the CD8^+^ T cell compartment was observed (Fig. 2f, *P* = 0.063, Welch’s t-test). The mean percentage of KLRG1 expressing CD8^+^ cells in the blood of our patient cohort was 32.7% and 52.9% in healthy individuals (*P* < 0.0001, Welch’s t-test; Fig. 2f), indicative of immunosenescence [19]. Furthermore, we observed significantly lower TIGIT expression on peripheral Tregs in NSCLC patients compared to healthy individuals (*P* = 0.001, Welch’s t-test, Fig. 2g). Since TIGIT has been implicated in enhancing the suppressive function and stability of regulatory T cells, particularly in inflammatory and tumor settings, this reduction may indicate a shift toward a less suppressive or destabilized peripheral Treg phenotype in NSCLC patients with brain metastases [20].

Taken together, this analysis shows a mixed phenotype, as potentially tumor-induced immunosuppressive molecule such as CD73 is upregulated in peripheral T cells, whereas others, such as TIGIT and KLRG1, are reduced in NSCLC BM patients.

### Peripheral blood immune profile of patients with oligometastatic disease

**Fig. 3:**
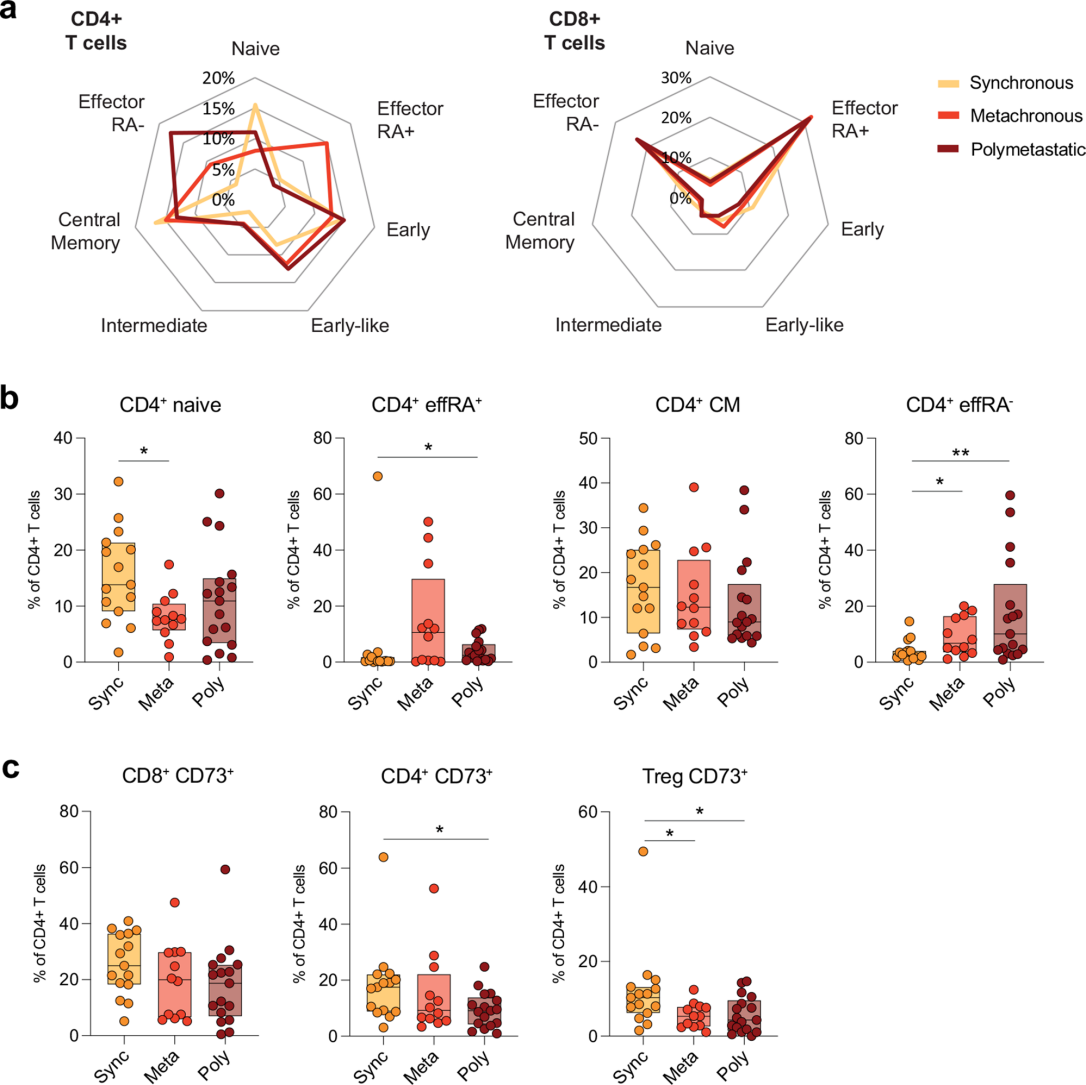
Flow cytometric immunophenotyping of T cell differentiation and CD73 expression in the different BM groups. **(a)** Spider plots of CD4 + and CD8 + differentiation phenotypes of peripheral blood in BM groups. Populations are defined by expressions of CD45RA, CD27, CD28, and CCR7 markers. **(b)** Box plots depicting expression of CD4 + naïve, TeffRA+, central memory, and TeffRA- (T_EM_) expressions between BM groups. **(c)** Box plots of CD73 expressions in CD8+, CD4+, and Treg cells between BM groups

Next we analyzed patient samples stratified according to their metastatic phenotype to further dissect relevant changes in the T cell compartment. The most intriguing findings were related to CD4^+^ T cell subtype differentiation. Supervised analyses revealed significant mean differences in the percentages of CD4^+^ T cell populations between the three brain metastatic groups, whereas no such differences were observed in CD8^+^ T cells (Fig. 3a). These CD4^+^ subtypes, which reflect the composition of differentiation and activation in the peripheral blood compartment and play crucial roles in mediating adaptive immunity, exhibited fewer CD4^+^ effector type RA^+^ cells (T_Eff_RA^+^) among oligo-synchronous patients compared to oligo-metachronous (*P* = 0.053, Wilcoxon test) and polybrain metastatic groups (*P* = 0.020, Wilcoxon test), respectively (Fig. 3a and b). Similarly, the median percentage of CD4^+^ effector type RA^-^ cells (T_EM_) was significantly lower in oligo-synchronous patients than in the other two groups (*P* = 0.021 and *P* = 0.002, respectively, Wilcoxon test), whereas, no significant differences were observed among the three brain metastatic groups in terms of CD4^+^ early, early like, intermediate, or central memory cells. Finally, the median percentage of CD4^+^ naïve cells was significantly higher in the oligo-synchronous group (*P* = 0.012, Wilcoxon test) than in the oligo-metachronous group (Fig. 3a and b). To assess whether pretreatment could have an impact on the T cell functional differentiation profiles of the BM groups, we compared all untreated and treated patients for checkpoint and cytokine production. CD39 and IL-10 in CD4^+^ T cells and TNF alpha from NK cells were significantly higher in treated patients compared to untreated (Supplementary Fig. 8), while no difference was observed in CD4^+^ IL-17 or any other checkpoint molecules. In addition, a comparison was made between untreated samples from oligo-synchronous and poly BM. The results of this analysis did not lead to an alteration in the significant differences observed. However, when only untreated oligo-synchronous samples were compared with pretreated oligo-metachronous samples, no significant differences were observed (Supplemental Figs. 9a-c). This shows that the differences CD4^+^ T cell populations was mainly group dependent with treatment not being a confounding factor except for possibly IL-10.

Next, the surface ectonucleotidases CD73 and CD39, which are activation/exhaustion markers, were assessed. CD73 expression was investigated in both CD4^+^ and CD8^+^ T cells and a significant mean difference was observed in CD4^+^ cells between the oligo-synchronous and poly BM groups (*P* = 0.022, Wilcoxon test), whereas no difference was observed for the CD8^+^ T cells (Fig. 3c). The observed difference in CD4^+^ was also significant for the subset of regulatory T cells (T_regs_) between the oligo-synchronous and poly BM groups (*P* = 0.023, Wilcoxon test) and between the oligo-synchronous and -metachronous BM groups (*P* = 0.016, Wilcoxon test) (Fig. 3c). No difference in the percentage of any of the T cell subgroups expressing CD39 was discerned between the patient groups.

**Fig. 4:**
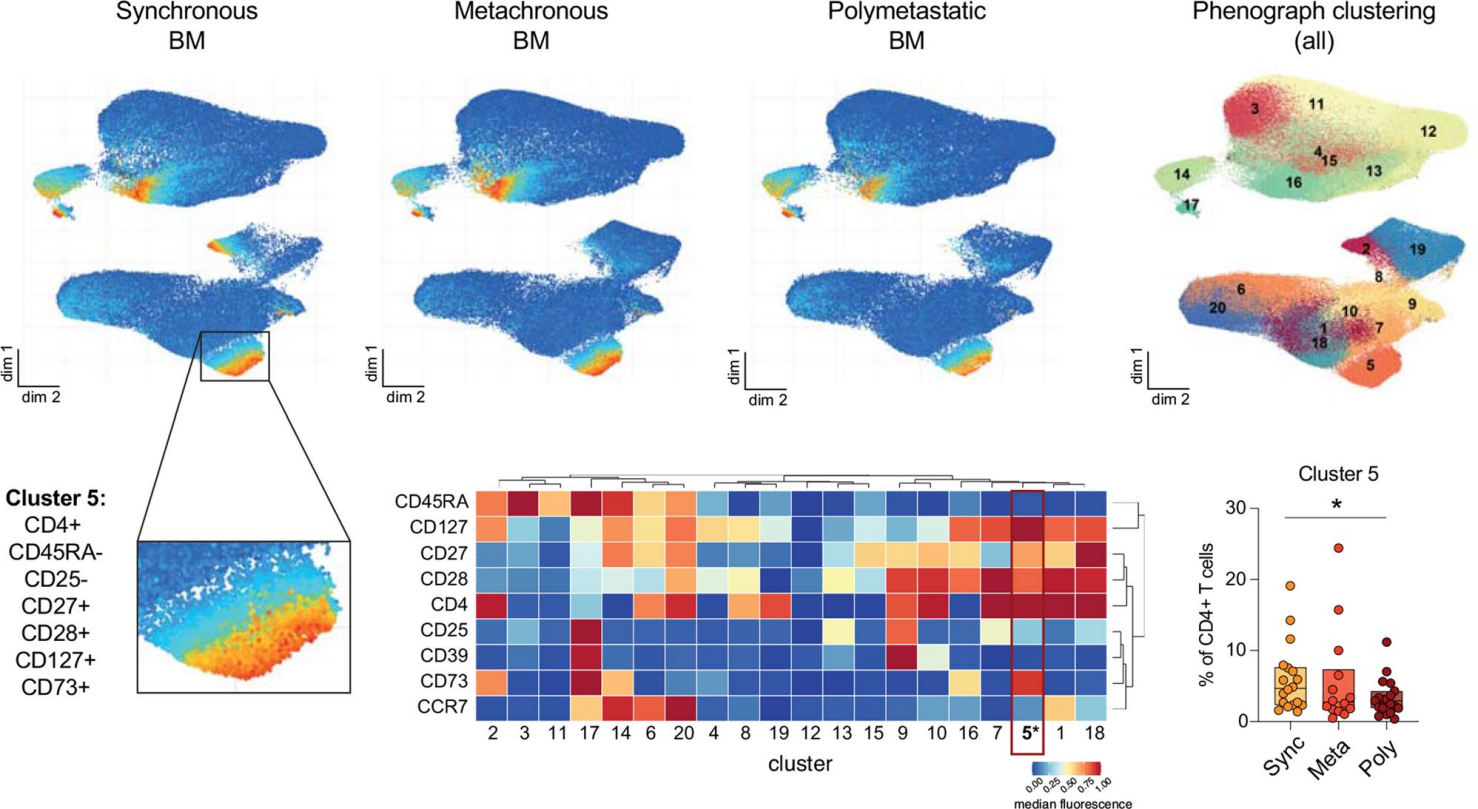
Phonograph clustering of different markers in the three BM groups combined, and individual UMAPs display CD73 expression in synchronous, metachronous, and polymetastatic BM. Heatmap and boxplot of markers expression in cluster 5: CD4+, CD27+, CD28+, CD127+, CD73 + and CD45RA-, CD25- T cells. Values describe population size as percentages. Sync: oligo-synchronous BM, Meta: oligo-metachronous BM, and poly: poly metastasis)

To further dissect the subpopulations, we performed an unsupervised UMAP analysis. The results indicate again a specific role for CD73 in the oligo-synchronous group, as shown by the difference in the percentage of CD73 expressing cells observed between the groups in cluster 5 and at the tip of cluster 2 (Fig. 4). The heat map shows that the cluster 5 mainly consisted of CD4^+^, CD27^+^, CD28^+^, CD127^+^, CD73^+^ and CD45RA^-^, CD25^-^ cells. These cells were significantly enriched in the oligo-synchronous group compared with the poly metastatic group (*P* = 0.044, t-test). These results point to the metastatic phenotype of oligo-synchronous metastasis being associated with altered CD4^+^ T cell differentiation.

## Discussion

Systemically, tumor cells and the peripheral immune system exhibit a mutual influence, resulting in what is termed a systemic immune environment [21]. Consequently, we conducted a comprehensive analysis of both the local and peripheral immunological characteristics of patients with NSCLC with varying brain metastatic patterns. To our knowledge, no previous studies have investigated the immune profiles among different brain metastatic patterns. Our analysis revealed a distinct augmentation of CD4^+^ T cells among the oligo-synchronous BM patients. This difference was particularly evident in terms of intra-tumoral CD4^+^ T cell infiltration.

In a first step, our multi-parametric immune phenotyping of peripheral blood identified several immune cell populations differentiating healthy controls from BM patients. Our investigation showed a significant increase in both CD4^+^ T_H_17 cells and elevated IL-17 cytokine production in the peripheral blood of BM patients compared to healthy individuals. The involvement of T_H_17 cells, characterized by production of IL-17, has been extensively studied in various cancer entities [22] with previous studies reporting higher T_H_17 lymphocytes in peripheral blood of patients with NSCLC [23, 24]. Our data, thus suggest an involvement of T_H_17-mediated responses in the immunopathology of NSCLC BM. In our study, we furthermore observed a significant upregulation of IL-10 producing CD4^+^ and CD8^+^ T cells in the BM patients, compared to healthy controls. This finding is consistent with the previously reported increase in IL-10 levels (partially alos due to treatment) in the peripheral blood of cancer patients, in which higher levels also predicted a worse outcome [25, 26]. Notably, the elevation of IL-10 in our cohort of NSCLC patients with BM complements our observations of an overall inflammatory phenotype in NSCLC, characterized by heightened levels of T_H_17 cells and IL-17. The concurrent upregulation of IL-10 and IL-17 underscores the interactive balance between pro- and anti-inflammatory responses in the immune system as modulated by cancer. In summary, this study revealed significant alterations in the peripheral immune system, particularly within the CD4^+^ T cell compartment, when comparing the global effects of NSCLC BM and healthy individuals.

When the patients were stratified into different metastatic phenotypes, specific alterations within CD4^+^ compartment in both the BM tissue and peripheral was observed for the oligometastatic group, wherea such distinction was not observed for CD8^+^ subtypes. Our analysis revealed a distinct augmentation of CD4^+^ T-cells among the oligo-synchronous BM patients. This difference was particularly noticeable in terms of intra-tumoral CD4^+^ T-cell infiltration. Similarly, in the peripheral blood, the CD4^+^ differentiation phenotypes discriminated the different metastatic phenotypes. This observation may suggest that metastatic patterns elicit different immune responses specifically in the peripheral CD4^+^ compartment. We observed a higher proportion of CD4^+^ naïve cells, whereas T_Eff_RA^+^ and T_Eff_RA^-^ cells were significantly less prevalent in the oligo-synchronous BM group. Naive T cells function in immune surveillance, circulating in the blood and responding to foreign antigens. Consequently, they differentiate into effector cells T_Eff_RA^+^ that eliminate or assist other immune cells in attacking the pathogen, or later into cells such as T_Eff_RA^-^, which subsequently perform the memory immune response when the infection recurs [27, 28]. Our results thus indicate that patients with oligo-synchronous BM exhibit a less activated immune system, suggesting that a brain metastasis alone may not evoke a strong immune response in the periphery, nonetheless permitting the outgrowth of a tumor in the brain.

Furthermore, our study demonstrated a general upregulation of CD73, again particularly on CD4^+^ T cells, in all BM patients compared to HD, with the most pronounced upregulation observed among oligo-synchronous BM patients. The unsupervised analysis revealed a distinct cell population in oligo-synchronous BM, comprised of CD4^+^, CD27^+^, CD28^+^, CD127^+^, CD73^+^ and CD25^-^ cells. These cells are likely memory CD4^+^ cells. Notably, Doherty and colleagues identified effector-memory CD73^+^ CD4^+^ T cells as T_H_17 cells [29], and Gourdin et al. demonstrated that CD73 identifies a subset of CD4 + effector T cells enriched with T_H_17 populations [30]. These findings are consistent with the higher T_H_17 frequency observed in our BM cohort. Beyond its well-characterized enzymatic function [31], CD73 functions as a lymphocyte differentiation antigen, suggesting involvement in lymphocyte maturation, development, and T cell activation. CD73 also serves as an adhesion molecule, facilitating the binding of lymphocytes to the endothelium [32–34]. While research on CD73 in the peripheral immune system is limited, its impact on tumor progression and anti-tumor responses in NSCLC has been observed in the TME [35, 36]. Given the multifaceted roles and mechanisms of CD73, careful interpretation of our results is warranted. Although the precise function of CD73 in BM-NSCLC requires further investigation, our data indicate significant differences among the different BM cohorts.

In general, the role of CD4^+^ T cells in immuno-oncology has gained increasing recognition [37]. Notably, CD4^+^ T cells, including T-helpers and Tregs, can affect metastatic progression independently of CD8^+^ T cells [38–40]. It has been proposed that peripheral CD4^+^ T cell differentiation patterns can independently predict tumor progression in NSCLC [41]. Our data further substantiates that this application also provides valuable information on the type of metastatic spread of BM patients. Given the highly specialized immune environment of the brain, data on CD4^+^ T cell composition in the brain tumor microenvironment is limited. A recent single-cell multi-omics analysis of different brain metastases indicated, similarly to our findings, that CD4^+^ T cells exhibit a significantly greater degree of phenotypic heterogeneity compared to CD8^+^ T cells [42]. Our findings, in conjunction with previous research, indicate a vital role of CD4^+^ T cells in mediating antitumor immunity in NSCLC BM, irrespective of CD8^+^ T cells.

Although our cohort size was limited, we conducted a comprehensive analysis of the peripheral and microenvironment immune landscape of NSCLC BM patients. We focused primarily on T cells; however, other immune cells likely influence BM formation. To more directly assess whether peripheral CD4 + T cell phenotypes reflect the immune landscape within brain metastases, a detailed characterization of intratumoral T cell subsets - including their functional states and spatial distribution - would be required. Such an investigation could yield important insights into how the peripheral immune system influences or reflects the degree of T cell infiltration into tumors. Future studies could build on our findings to explore the dynamic interplay between peripheral immune phenotypes and intratumoral immune architecture. Additionally, a comparison with non-BM NSCLC patients would be desirable; however, given the heterogeneous metastatic patterns in such cases and the frequently missing information about micrometastases in the brain in asymptomatic patients, such comparative analyses are impeded. Moreover, future studies need to elucidate the biological role of these identified differences, especially in driving oligometastatic disease.

In conclusion, our data demonstrate that NSCLC BM patients exhibit mainly a skewed systemic CD4^+^ T cell phenotype compared to healthy individuals, defined by the T_H_17/IL-17 axis. Additionally, a specific immune profile was identified separating especially the oligo-synchronous BM from the other groups in both peripheral blood and brain tumor microenvironment again defined by specific CD4^+^ T cell populations defined by in general a less activated immune system but activation of CD73.

## Electronic supplementary material

Below is the link to the electronic supplementary material.

Supplementary Material 1

## Data Availability

No datasets were generated or analysed during the current study.
